# The Impact of Vatinoxan on the Concentrations of Medetomidine, Midazolam, and Fentanyl in Central Nervous System After Subcutaneous Co‐Administration in Rats

**DOI:** 10.1111/jvp.13514

**Published:** 2025-04-21

**Authors:** Juhana Honkavaara, Emily Lindh, Anna Meller, Karoliina Alm, Marja R. Raekallio, Pernilla Syrjä

**Affiliations:** ^1^ Faculty of Veterinary Medicine, Department of Equine and Small Animal Medicine University of Helsinki Helsinki Finland; ^2^ Laboratory Animal Centre University of Helsinki Helsinki Finland; ^3^ Faculty of Veterinary Medicine, Department of Veterinary Biosciences University of Helsinki Helsinki Finland

**Keywords:** fentanyl, medetomidine, midazolam, rat, vatinoxan

## Abstract

Our aim was to investigate whether vatinoxan, a peripherally acting alpha_2_‐adrenoceptor antagonist, would affect the concentrations of medetomidine, midazolam, and fentanyl in the central nervous system after subcutaneous co‐administration. Twelve healthy male Wistar rats, aged between 13 and 15 weeks, were used in this study. The animals received one of two subcutaneously administered treatments: medetomidine 0.25 mg/kg, midazolam 2 mg/kg, and fentanyl 0.01 mg/kg (MMF) or MMF with 5 mg/kg of vatinoxan (MMF‐V). 15 min later, the sedated rats were humanely euthanized with intravenous pentobarbital. Plasma and tissue, including aliquots of the cortex, thalamus, pons, and lumbar spinal cord, were harvested and analyzed for drug concentrations. The treatments were compared with Bonferroni corrected *t*‐tests after one‐way analysis of variance. The concentrations of medetomidine (144 ± 19.4 vs. 107 ± 13.1 ng/g [mean ± 95% confidence interval]) (*p* = 0.04) and fentanyl (2.3 ± 0.2 vs. 1.7 ± 0.3 ng/g) (*p* = 0.04) in the cortex were significantly higher in the rats administered MMF‐V. Similarly, cortex: plasma drug concentration ratios were significantly higher for medetomidine, midazolam, and fentanyl after MMF‐V (*p* < 0.001 for all). The results confirm that vatinoxan increases early cortical exposure to subcutaneously co‐administered medetomidine and fentanyl.

## 1 Introduction

To produce an effect at the level of the central nervous system, efficacious drug concentrations at the site of action are required (Doze et al. 1989; Guo et al. 1996). For sedative drugs used in veterinary medicine, this often involves an absorption phase after extravascular delivery followed by distribution across the blood–brain barrier. Previously published evidence suggests that alpha_2_‐adrenoceptor agonists, which are commonly used to produce sedation both alone and in combination with other drugs, impair drug absorption after extravascular, parenteral administration by producing local vasoconstriction at the injection site (Restitutti et al. 2017; Kallio‐Kujala et al. 2018). This effect might lead to extreme delays in reaching maximal plasma concentrations, particularly if the drugs are deposited in poorly perfused compartments, as reported for subcutaneously administered medetomidine in isoflurane‐anesthetized rats (Kint et al. 2020). Furthermore, the signature surge in systemic vascular resistance produced by alpha_2_‐adrenoceptor agonists reduces drug distribution (Honkavaara et al. 2012; Pypendop et al. 2016; Bennett et al. 2017). Consequently, the onset of their sedative effect, and of other co‐administered centrally acting drugs, becomes delayed and more vulnerable to individual variation, both clinically unfavorable consequences (Restitutti et al. 2017; Kallio‐Kujala et al. 2018; Lindh et al. 2024). Vatinoxan is an alpha_2_‐adrenoceptor antagonist that does not appear to markedly permeate the mammalian blood–brain barrier, thus selectively attenuating the peripheral effects of simultaneously administered alpha_2_‐adrenoceptor agonists (Clineschmidt et al. 1988, Honkavaara et al. 2020). It has recently become commercially available in a fixed combination with medetomidine. By preventing the agonist‐induced vasoconstriction at both the systemic and local level (i.e., extravascular administration site), vatinoxan has been suggested to both enhance the absorption rate and increase the volume of distribution of co‐administered drugs (Bennett et al. 2017; Kallio‐Kujala et al. 2018; Turunen et al. 2020). In view of this, the lead time to sedation was in fact reduced by the addition of vatinoxan in both dogs and rats administered intramuscular or subcutaneous combinations of medetomidine, midazolam, and an opioid, respectively (Kallio‐Kujala et al. 2018; Lindh et al. 2024). Similar outcomes were also reported in cats after intramuscular co‐administration of vatinoxan and dexmedetomidine (Honkavaara et al. 2017, Pypendop et al. 2017b). However, plasma drug concentrations and clinical effects provide only indirect evidence of the amount of drug reaching the central nervous system. Therefore, we aimed to investigate whether vatinoxan would, in fact, increase the concentration of sedative‐analgesics in the central nervous system. We hypothesized that at a time of early sedation, the central nervous system of rats receiving vatinoxan would have greater exposure to medetomidine, midazolam, and fentanyl after subcutaneous co‐administration in a single syringe.

## 2 Material and Methods

### 2.1 Animals

Twelve healthy male, Wistar rats (Envigo, Horst, Netherlands) aged between 13 and 15 weeks and weighing 360–422 g were used in this study. The rats were housed at the Laboratory Animal Center of the University of Helsinki, Finland. They were kept in groups of three, in individually ventilated cages (Blue line 1500 U for Rats, Techniplast, Italy) under regulated temperature (21°C ± 1°C), humidity (55% ± 10%) and 12/12 h light–dark cycles. The rats were fed a standard rodent diet (Teklad 8460, Harlan Teklad, Inotiv, USA) and had free access to purified water at all times. The animals were acclimatized to human handling and had weekly access to an “activity‐center”. Nine of the rats had previously been used in a cross‐over study, where they had received repeated subcutaneous doses of medetomidine, midazolam and vatinoxan. A minimum of 10 days between the present study and any previous drug administration was allowed for those animals. The study was approved by the National Animal Experimentation Board of Finland (permit ID ESAVI/39801/2023). The license fulfills all requirements of the European Union legislation and the updated ARRIVE 2.0 guidelines (du Sert et al. 2020).

### 2.2 Study Design and Protocol

The animals received one of two treatments, administered subcutaneously into the neck scruff: medetomidine 0.25 mg/kg (Dorbene 1 mg/mL, SIVA Laboratories) + midazolam 2 mg/kg (Midazolam 5 mg/mL, Hameln Pharma GmbH) + fentanyl 0.01 mg/kg (Fentanyl 50 μg/mL, Hameln Pharma GmbH) (MMF) with or without vatinoxan 5 mg/kg (MMF‐V). For MMF‐V, a commercially available combination of medetomidine and vatinoxan (Zenalpha 0.5/10 mg/mL, Vetcare Ltd.) was used. Sterile 0.9% saline was added to MMF to achieve an identical injectate volume with MMF‐V. The person administering the drugs was blinded to the treatment. 10 min after drug administration, the tail vein was cannulated with a 25 G intravenous catheter (Versatus, Terumo BCT, CO, USA). If a rat required manual restraint for cannulation, the animal was excluded from the study, and the next rat received the same treatment until successful. The excluded rats were humanely euthanized with 50 mg/kg of intraperitoneal pentobarbital. Hence, the order of treatments was not randomized but followed a 1:1 ratio. Once cannulated, 0.5 mg of indocyanine green (ICG) dissolved in 0.5 mL of sterile saline was administered as an IV bolus. 5 min later (i.e., 15 min from drug injection) the rats were administered a 25 mg/kg bolus of intravenous pentobarbital. Once the loss of the pedal withdrawal reflex was confirmed, cardiocentesis was performed to obtain a 2 mL blood sample that was transferred into a tube with ethylenediaminetetraacetic acid (EDTA). The rats were then immediately decapitated, the skull opened, and the brain removed for sampling. Fresh tissue samples were obtained from pre‐selected areas of the central nervous system: left parietal cortex, thalamus, pons, and spinal cord at the level of the lumbar plexus. The postmortem tissue sampling was performed by a board certified veterinary pathologist (PS) who was unaware of the treatments. The meninges were not removed from cortical, pontine, and spinal cord samples. The thalamic samples consisted solely of brain tissue (interthalamic adhesion). Cerebrospinal fluid was not dried out from the samples. The tissue samples were immediately frozen in liquid nitrogen and stored in −80°C until thawed for analyses of tissue drug concentrations. The EDTA blood samples were centrifuged without delay (3000 rpm for 10 min), plasma transferred to cryotubes which were capped and dipped in liquid nitrogen, put in dry ice, and transferred to −80°C until thawed for analysis of plasma drug concentrations.

### 2.3 Plasma and Tissue Drug Concentration Analyses

Concentrations of medetomidine, vatinoxan, midazolam, and fentanyl were quantitatively determined by LC/MS/MS with a Waters Acquity UPLC liquid chromatograph coupled with a Waters XEVO‐TQ‐Absolute triple quadrupole (Waters, Milford MA, USA). Prior to analysis, tissue samples were homogenized in 150 mM phosphate buffered saline (PBS, pH 7.4) using an Omni Bead Ruptor bead homogenizer to a weight‐to‐volume ratio of 1:4 (e.g., 100 mg of tissue was homogenized in 400 μL of PBS) for a final dilution factor of 5. Samples were prepared for analysis with protein precipitation: 100 μL of each homogenate was precipitated with 300 μL of cold acetonitrile (containing 100 ng/mL of phenacetin as an internal standard). After mixing for 3 min on a tabletop shaker, the sample was centrifuged at 2272 × *g* for 20 min. The supernatant was transferred to an analytical plate, diluted 1:1 with ultrapure water, and submitted to analysis with LC–MS/MS.

Standard samples were prepared by spiking rat plasma and whole brain homogenate to concentrations of 0.001–200 ng/mL for fentanyl and 0.005–1000 ng/mL for the other compounds. Quality control (QC) samples were prepared by spiking blank rat plasma and whole brain homogenate to concentrations of 0.75, 7.5, 75, and 750 ng/mL of fentanyl, or 3, 30, 300, and 3000 ng/mL for the rest of the compounds, in duplicate. Standard and QC samples were prepared for analysis identically to the study samples. The analysis of all studied compounds was performed for both plasma and whole brain homogenate with the same analytical method. The chromatography consisted of a gradient elution of 0.1% formic acid and acetonitrile on a Waters Acquity UPLC BEH C8 (2.1 × 50 mm, 1.7 μm) column equipped with a pre‐column (Waters, Milford, MA, USA).

The analysis was run at a constant temperature of 35°C, with a flow rate of 0.5 mL/min and the chromatography consisted of a gradient of acetonitrile that ranged from 5% to 70% in 2.5 min rising to 95% in the following minute before dropping straight to 5% ending with a 2‐min equilibration phase before the next sample injection. The compounds were quantitatively monitored in ESI+ mode with the following MRM reactions: 337 > 188 for fentanyl, 201 > 95 for medetomidine, 326 > 291 for midazolam, 419 > 281 for vatinoxan, and 180 > 110 for phenacetin. The retention times for the analytes were 1.92, 1.78, 1.91, 1.52, and 1.82 min, respectively. For fentanyl, the calibration curve was fitted from 0.2 to 20 ng/mL in plasma and 0.02 to 10 ng/mL in tissue homogenate. The accuracy for all calibrators ranged between 88.7% and 106.9% and between 70.8% and 93.1% for the QC samples. For medetomidine, the calibration curve was fitted from 1.0 to 1000 ng/mL in plasma and 0.2 to 50 ng/mL in tissue homogenate. The accuracy for all calibrators ranged between 81.3% and 112.9% and between 70.8% and 112.7% for the QC samples. For midazolam, the calibration curve was fitted from 1.0 to 1000 ng/mL in plasma and 1 to 200 ng/mL in tissue homogenate. The accuracy for all calibrators ranged between 84.4% and 108.5% and between 93.7% and 138.2% for the QC samples. For vatinoxan, the calibration curve was fitted from 1.0 to 1000 ng/mL in plasma and 1 to 500 ng/mL in whole brain homogenate. The accuracy for all calibrators ranged between 87.9% and 112.0% and between 77.4% and 114.5% for the QC samples. As the analytical run consisted of short sample series containing only two series of calibration runs, Snedecor's F distribution was calculated to determine analytical precision. The Snedecor's percentage for all analytes and both matrices ranged between 2.4% and 9.4%. All analytes were analyzed in one same analytical run in both plasma and the central nervous system samples. The respective LLD and LLOQ in plasma were for medetomidine 0.1 and 1 ng/mL, for midazolam 0.1 and 1 ng/mL, for fentanyl 0.1 and 0.2 ng/mL, and for vatinoxan 0.1 and 1 ng/mL. In whole brain homogenate the respective LOD and LLOQ values were, for medetomidine 0.1 and 0.2 ng/mL, for midazolam 0.1 and 1 ng/mL, for fentanyl 0.2 and 1 ng/mL, and for vatinoxan < 0.02 and 0.02 ng/mL. Despite multiple efforts, the analysis of ICG concentrations in plasma failed to pass quality control requirements for unknown reasons.

### 2.4 Statistical Analysis

The sample size was calculated based on an expected 50% difference in tissue drug concentrations between treatments and a 20% standard deviation within treatments, suggesting that six animals per group would be sufficient with a power of 80% and an alpha level of 0.017. We further estimated that the power would suffice to detect a significant difference in tissue: plasma drug concentrations between treatments. The distribution of the data was assessed with a Shapiro–Wilk's test, followed by a one‐way analysis of variance and Bonferroni (threefold) corrected post hoc Student's *t*‐tests for comparisons between treatments. Significance was thus accepted with an adjusted alpha level set ≤ 0.05. Data is presented as mean ± 95% confidence intervals.

## 3 Results

Three rats did not become sedated enough to be cannulated without restraint 10 min after drug administration (two after MMF and one after MMF‐V). They were replaced by three male Wistar rats of a similar age and weight but who had not been previously sedated. All included rats were subjectively assessed as deeply sedated 10 min after drug administration and no rat reacted to the placement of the venous catheter into a tail vein. The analysis for plasma ICG concentrations was not stable and its concentrations for both plasma and tissue were excluded from the results.

The drug concentrations in plasma and central nervous tissue are presented in Table 1. The concentrations of medetomidine (*p* = 0.04) and fentanyl (*p* = 0.004) in the parietal cortex were significantly higher in rats that received MMF‐V. The drug concentration ratios for cortex: plasma were significantly higher for medetomidine (*p* < 0.001), fentanyl (*p* < 0.001) and midazolam (*p* < 0.001) in the MMF‐V treatment group.

**TABLE 1:** Drug concentrations in plasma and central nervous system and their ratios from rats treated 15 min earlier with subcutaneous medetomidine, midazolam, and fentanyl (MMF) or MMF with vatinoxan. Data is expressed as mean ± 95% confidence interval.

	Medetomidine		Midazolam		Fentanyl		Vatinoxan
	MMF	MMF‐V	MMF	MMF‐V	MMF	MMF‐V	MMF‐V
Concentration							
Plasma (ng/mL)	29.9 ± 7.2	18.4 ± 3.2	165 ± 46	97 ± 17	0.7 ± 0.1	*0.4 ± 0.1*	645 ± 186
Cortex (ng/g)	107 ± 13.1	*144 ± 19.4*	320 ± 92	446 ± 53	1.7 ± 0.3	*2.3 ± 0.2*	19 ± 6
Thalamus (ng/g)	89.3 ± 27.4	101 ± 43.5	322 ± 96	302 ± 142	1.5 ± 0.5	1.6 ± 0.4	30 ± 16
Pons (ng/g)	58.6 ± 18.3	65.2 ± 18.1	214 ± 59	233 ± 81	1.3 ± 0.2	1.1 ± 0.2	16 ± 6
Lumbar spinal cord (ng/g)	44.5 ± 15.2	60.8 ± 15.3	205 ± 59	283 ± 97	1.0 ± 0.2	1.6 ± 1.1	36 ± 14
Ratio							
Cortex to plasma	3.8 ± 0.7	*8.0 ± 1.2*	2.0 ± 0.6	*4.7 ± 0.7*	2.6 ± 0.5	*5.6 ± 1.0*	0.03 ± 0.01
Thalamus to plasma	3.2 ± 1.1	5.2 ± 1.5	2.2 ± 0.9	2.9 ± 0.9	2.1 ± 0.4	*4.1 ± 1.0*	0.05 ± 0.02
Pons to plasma	2.0 ± 0.4	*3.5 ± 0.6*	1.3 ± 0.3	2.5 ± 0.9	2.1 ± 0.5	2.7 ± 0.4	0.03 ± 0.01
Lumbar spinal cord to plasma	1.4 ± 0.2	*3.4 ± 1.1*	1.3 ± 0.3	3.1 ± 1.3	1.6 ± 0.4	3.8 ± 2.3	0.05 ± 0.01

*Note*: Significantly different (*p* < 0.05) from MMF when italicized.

## 4 Discussion

This study is the first to confirm that vatinoxan, a peripherally selective alpha_2_‐adrenoceptor antagonist, increases the cortical exposure to medetomidine and fentanyl after subcutaneous co‐administration. Moreover, the cortex: plasma concentration ratios for medetomidine, midazolam, and fentanyl in rats receiving vatinoxan were effectively doubled at 15 min after injection. The results offer a plausible explanation of why the onset of central effects is shortened by the addition of vatinoxan, which, by mitigating the vasoconstrictive effects of the agonist, allows for more efficient disposition of simultaneously delivered drugs (Lindh et al. 2024).

Interestingly, in this study, the concentrations of medetomidine, midazolam, and fentanyl in plasma appeared lower in the presence of vatinoxan, reaching statistical significance for fentanyl. This outcome seems in conflict with reported outcomes in dogs and cats, where vatinoxan increased concentrations of co‐administered drugs in plasma during early absorption after intramuscular co‐administration (Restitutti et al. 2017; Pypendop et al. 2017b; Kallio‐Kujala et al. 2018). In view of that, each factor affecting plasma drug concentrations should be considered within the context of a predominant absorption phase. First, more complete absorption would increase the amount of drug entering the central compartment, resulting in higher measured plasma drug concentrations. On the other hand, greater distribution of drugs into tissues from the central compartment reduces the concentration of drugs in plasma. Furthermore, any perfusion‐dependent step in biotransformation would likely be negatively affected by medetomidine‐induced vasoconstriction and decreased cardiac output. Therefore, the addition of vatinoxan is likely to increase the overall rate of phase I and II reactions in, for example, the liver, thus decreasing the amount of the parent compound in blood. To further complicate interpretation of plasma drug concentrations, vatinoxan expands the circulatory volume (i.e., volume of the central compartment) compared to animals receiving an alpha_2_‐adrenoceptor agonist alone (Pypendop et al. 2016). This effect, again mediated by vasodilation and coupled with lower systemic blood pressure, would favor hydrostatic fluid shifts from tissues towards plasma in vatinoxan‐receiving animals, leading to a higher degree of relative hemodilution. Consequent decreases in measured plasma drug concentrations would be expected. In contrast, further hemoconcentration would be anticipated in medetomidine‐treated animals due to the massive diuresis and subsequent water loss induced by alpha_2_‐adrenoceptor agonists, an effect observed in dexmedetomidine‐treated rats within 15 min and an outcome effectively blocked by vatinoxan (Horváth et al. 1996; Lindh et al. 2024). Last, although vatinoxan has not been reported to significantly affect plasma protein content in other species such as cats (Pypendop et al. 2017a), potentially altering the disposition of highly protein‐bound drugs, direct evidence in rats is not available to the best of our knowledge. Overall, simply by preventing the characteristic vasoconstriction produced by alpha_2_‐adrenoceptor agonists, vatinoxan probably influenced a myriad of events simultaneously affecting both plasma and tissue drug concentrations. Thus, it is worth stating the obvious that drawing far‐reaching conclusions from drug concentrations obtained at single time‐points outside steady‐state conditions is hazardous at best. The factors affecting drug disposition are complex and often overlapping, and interpreting the plasma drug concentrations from the present study could easily have led the authors to suggest that absorption appeared greater after MMF. However, lower plasma drug concentrations here were associated with higher drug exposure within the central nervous system—and vice versa—suggesting clear differences in distribution between the treatments at the time of sample collection. Species differences in, for example, drug effects, circulatory times, and metabolic rates are likely factors contributing to the discrepancies between previous studies and the present results.

Apart from vatinoxan, the drug concentrations appeared higher in the cortical and thalamic tissue compared to the pons or lumbar spinal cord. The relevance of this finding is unclear and since our main aim was to study the impact of vatinoxan, only descriptive statistics were performed with no effort to test any within‐treatment hypotheses. The regions of interest were chosen to represent relevant areas related to consciousness (cortex), an epicenter of alpha_2_‐adrenoceptor‐mediated activity (thalamus), autonomous regulation (pons) and a distal part of the central nervous system for its antinociceptive, pharmacological interest (lumbar spinal cord). That said, differences in drug concentrations were likely to represent dissimilarities in perfusion rather than their sites of action. In a previous study in dogs, the difference between concentrations of dexmedetomidine in brain and lumbar spinal cord was less obvious, perhaps reflecting more complete distribution as the drugs were administered intravenously 20 min a priori (Honkavaara et al. 2020).

The central nervous system: plasma vatinoxan concentration ratios were similar with previous reports in rats, dogs, and sheep (Clineschmidt et al. 1988; Honkavaara et al. 2020; Adam et al. 2021). It remains to be elucidated whether the amount of vatinoxan that does penetrate into the central nervous system is sufficient to have any clinically relevant effect. Hector et al. (2017) reported that vatinoxan did increase the minimal alveolar concentration of sevoflurane in dogs but were unable to suggest a definite explanation for their observation. Presently, there are no clinical indications for vatinoxan as monotherapy, and its central impact in the presence of agonist drugs appears negligible. Future studies are, however, warranted to investigate its later disposition into the central nervous system as all efforts to date have focused on relatively early time‐points after administration.

Several limitations need to be considered when interpreting the present results. Most importantly, only a single time‐point was available due to the terminal nature of the study. In the authors' experience, most rats administered subcutaneous MMF become adequately sedated within 15 min, whereas with MMF‐V sedation appears to occur a few minutes earlier (Lindh et al. 2024). An argument could be made that if more time were allowed to reach peak sedation, differences in tissue drug concentrations between treatments would have been less obvious. Similarly, selecting an earlier time‐point might have produced greater differences. While we acknowledge this limitation, a single time‐point had to be selected to test the hypothesis, and we were not willing to sacrifice an excessive number of animals required to produce a more comprehensive temporal understanding of how the plasma and tissue drug concentrations evolved over time. Also, the lack of randomization could be considered a flaw since it inherently suggests subject selection. However, since we expected more sedation failures with MMF, randomization would potentially have led to a drastically skewed data set and potentially required even more animals to be used to successfully reach sufficient statistical power. Furthermore, tissue sample contamination by blood was allowed as the authors were not willing to perform circulatory perfusion in live animals due to ethical concerns. Of the selected regions of interest, the parietal cortex was likely the best perfused. Since the drug concentration in the cortex were approximately five‐fold to plasma, the lack of exsanguination probably had only a minor effect on tissue drug concentrations. Initially, the rats were administered ICG in an effort to calculate and correct for the amount of residual blood in the tissue samples, since the volume of distribution of ICG in non‐hepatic tissues is practically zero (Center et al. 1983). Unfortunately, plasma ICG concentration analysis failed quality standards and all ICG data had to be rejected. Last, the sample size was small and consisted only of healthy, pain‐free, male animals, which needs to be taken into consideration when construing the outcome of this study.

In conclusion, vatinoxan increased the early concentrations of medetomidine and fentanyl in the central nervous system after subcutaneous co‐administration to male Wistar rats. The impact of vatinoxan was most obvious in the cortical tissue. The results point towards a mechanism by which vatinoxan decreases the lead time to sedative effects by simultaneously administered drugs.

## Author Contributions

**Juhana Honkavaara:** study design, data collection, data analysis, manuscript preparation. **Emily Lindh:** data collection, manuscript review. **Anna Meller:** data collection, manuscript review. **Karoliina Alm:** data collection, manuscript review. **Marja R. Raekallio:** study design, manuscript review. **Pernilla Syrjä:** study design, data collection, manuscript review.

## Ethics Statement

The authors confirm that the ethical policies of the journal, as noted on the journal's author guidelines page, have been adhered to and the appropriate ethical review committee approval has been received. The authors confirm that they have adhered to European standards for the protection of animals used for scientific purposes. The study was approved by the National Animal Experimentation Board of Finland (permit number ESAVI/39801/2023).

## Conflicts of Interest

Dr. Honkavaara has received funding in the past from Vetcare Ltd., the company that produces the commercially available medetomidine‐vatinoxan preparation Zenalpha. The other authors declare no conflicts of interest.

## Data Availability

The data that support the findings of this study are available from the corresponding author upon reasonable request.
